# CPF impedes cell cycle re‐entry of quiescent lung cancer cells through transcriptional suppression of FACT and c‐MYC

**DOI:** 10.1111/jcmm.14897

**Published:** 2020-01-20

**Authors:** Ling Bi, Chanlu Xie, Lijing Jiao, Shenyi Jin, Su Su Thae Hnit, Yao Mu, Yilun Wang, Qian Wang, Guangbo Ge, Yaqiao Wang, Xiaodong Zhao, Xinglong Shi, Yani Kang, Paul De Souza, Tao Liu, Jia Zhou, Ling Xu, Qihan Dong

**Affiliations:** ^1^ Department of Oncology Yueyang Hospital of Integrated Traditional Chinese and Western Medicine Shanghai University of Traditional Chinese Medicine Shanghai China; ^2^ School of Science and Health National Institute of Complementary Medicine Western Sydney University Penrith South NSW Australia; ^3^ Chinese Medicine Anti‐cancer Evaluation Program Greg Brown Laboratory Central Clinical School and Charles Perkins Center The University of Sydney Sydney NSW Australia; ^4^ Department of Endocrinology Royal Prince Alfred Hospital Sydney NSW Australia; ^5^ Origins of Cancer Program Centenary Institute Camperdown NSW Australia; ^6^ Sydney Medical School the University of Sydney Sydney NSW Australia; ^7^ Institute of Interdisciplinary Integrative Medicine Research Shanghai University of Traditional Chinese Medicine Shanghai China; ^8^ Shanghai Center for Systems Biology Shanghai Jiao Tong University Shanghai China; ^9^ School of Biomedical Engineering Shanghai Jiao Tong University Shanghai China; ^10^ School of Medicine Western Sydney University Campbelltown NSW Australia; ^11^ Children’s Cancer Institute Australia for Medical Research Sydney NSW Australia; ^12^ Center for Childhood Cancer Research UNSW Medicine Sydney NSW Australia; ^13^ Department of Thoracic Surgery Yueyang Hospital of Integrated Traditional Chinese and Western Medicine Shanghai University of Traditional Chinese Medicine Shanghai China

**Keywords:** c‐MYC, G_0_ cell cycle re‐entry, lung cancer, structure‐specific recognition protein 1, suppressor of Ty homolog‐16

## Abstract

Blockade of cell cycle re‐entry in quiescent cancer cells is a strategy to prevent cancer progression and recurrence. We investigated the action and mode of action of CPF mixture (*Coptis chinensis, Pinellia ternata* and *Fructus trichosanthis*) in impeding a proliferative switch in quiescent lung cancer cells. The results indicated that CPF impeded cell cycle re‐entry in quiescent lung cancer cells by reduction of FACT and c‐MYC mRNA and protein levels, with concomitant decrease in H3K4 tri‐methylation and RNA polymerase II occupancy at FACT and c‐MYC promoter regions. Animals implanted with quiescent cancer cells that had been exposed to CPF had reduced tumour volume/weight. Thus, CPF suppresses proliferative switching through transcriptional suppression of FACT and the c‐MYC, providing a new insight into therapeutic target and intervention method in impeding cancer recurrence.

## 1 INTRODUCTION

With improvements in treatment, a growing list of cancers has become chronic diseases. In 2016, there were 14 million cancer survivors in the United States alone but many of them could later die from cancer recurrence. According to GLOBOCAN 2018, global cancer deaths could reach 9.6 million in 2018.^1^ Hence, prevention of cancer recurrence is a major concern facing the growing population of cancer survivors, given the reality that no secondary recurrence prevention method is available after completion of primary treatment.

G_0_ phase is a cellular state in which cells are in reversible cell cycle arrest. To multicellular organisms, the G_0_ state is necessary for development as cells at different stages of development lineage, including stem cells, slow‐cycling cells and differentiated mature cells, all adopt the G_0_ state temporally or spatially.^2^, ^3^ Cancer cells can also share the G_0_ state and are also known as dormant or quiescent cancer cells.^4^ Together with insufficient angiogenesis and immune surveillance, quiescent cancer cells are one mechanism explaining clinically observed cancer dormancy.^4^ The presence of dormant cancer cells is a two‐edge sword. While it is beneficial to a multicellular organism for cancer cells with uncontrolled proliferation to be halted, the same cancer cells in quiescence can survive most chemo‐ and radiotherapy and may, with time, acquire additional mutations and gain metastatic potential as they re‐enter the cell cycle.^5^ The therapeutic strategy of eliminating G_0_ cancer cells or promoting cell cycle re‐entry by quiescent cancer cells is regarded as risky, as treatment efficacy in the G_0_ phase is uncertain, and surviving cells may be selected out for more aggressive characteristics. Also, there is no assurance that antiproliferative drugs will be sufficiently effective to eliminate the reactivated G_0_ cancer cells.^5^ Considering that proliferation of G_0_ cancer cells upon cell cycle re‐entry can simply replace the eradicated cancer cells during primary treatment,^6^, ^7^ the development of a therapeutic strategy aimed at impeding cell cycle re‐entry by quiescent cancer cells is of importance. A major prerequisite to be able to implement this strategy is to identify the therapeutic target responsible for the proliferative switch and to establish an effective and safe method capable of exerting action on the therapeutic target.

Our knowledge of the mechanisms underlying cell cycle re‐entry from a G_0_ state is growing. Apart from cyclin‐dependent kinase inhibition,^8^ histone modification, RNA interference and autophagy are all implicated in the proliferative switch from quiescent state.^3^, ^9^, ^10^, ^11^, ^12^, ^13^ Facilitates Chromatin Transcription (FACT) is a member of histone chaperone family and consists of subunits of structure‐specific recognition protein 1 (SSRP1) and suppressor of Ty homolog‐16 (SPT16). In the nucleosome, FACT facilitates the passage of DNA and RNA polymerase by temporal eviction of histones.^14^ FACT then promotes the deposition of histones to re‐establish the nucleosome.^14^ However, against intuition, genetic silencing of the histone chaperones affects only about 2% of gene transcription and elongation in lung cancer cells.^15^ Also, FACT is aberrantly overexpressed in cancers of breast, lung, pancreas and brain.^16^, ^17^ Recently, we have shown that FACT mRNA and protein levels oscillate between the quiescent and proliferative state, and FACT is necessary and sufficient in the proliferative switch of quiescent lung cancer cells through transcriptional regulation of c‐MYC, which in turn influence cyclin‐dependent kinase inhibitor p27 and its regulatory proteins such as SKP2.^18^

Ideally, in cancer patients who have completed intensive treatment, sometimes with considerable toxicity, any subsequent treatment should be not only effective but possess few side effects, especially if quiescent cancer cells are to be maintained in the G_0_ phase for a considerable period. Based on the principles of effective therapy, minimal toxicity and economical use, as well as the notion of ‘an old drug for anew use’, we have investigated Chinese herbal medicines for preventing cell cycle re‐entry of quiescent lung cancer cells. The ancient book ‘Treatise on Miscellaneous Diseases’ written by Zhang Zhongjing (AD 150‐219) is the first known complete collection of Chinese medicine prescriptions.^19^ Known as the ‘ancestral book’ in China, the prescriptions are respected as ‘Kampo’ (Han dynasty prescriptions) in Northeast Asia and are still used in universities of Chinese medicine. CPF (Chinese name: Xiao‐Xian‐Xiong Tang) was first described in the ‘Treatise on Miscellaneous Diseases’ and consists of three herbs: *Coptis chinensis, Pinellia ternata* and *Fructus trichosanthis* (Figure S1A). Based on the HPLC results, of the 63 compounds identified, 43 were from Coptis chinensis (Figure S1B,C and Table S1). According to the theory of traditional Chinese medicine, CPF is used for treating diseases of the respiratory system, including pneumonia, asthma and pulmonary fibrosis in China. Here, we present evidence that CPF is able to impede the proliferative switch of quiescent lung cancer cells by transcriptional suppression of FACT and c‐MYC genes.

## 2 MATERIALS AND METHODS

### 2.1 Cell lines

The lung cancer cell lines A549 (Cat. #: CCL‐185) and H1975 (Cat. #: CRL‐1435) and normal bronchial epithelial cell lines 16HBE (Cat. #: CCL‐2741) and BEAS‐2B (Cat. #: CCL‐9609) were obtained from ATCC and grown in RPMI 1640 supplemented with 10% v/v foetal bovine serum (AusGeneX), penicillin (100 U/mL) and streptomycin (100 μg/mL). The cells were cultured at 37°C with 5% CO_2_/95% air.

### 2.2 CPF preparation

CPF consists of *Coptis chinensis, Pinellia ternata* and *Fructus trichosanthis*. All the herbs obtained from Huayu Pharmacy Company were certificated based on the authentication by Chinese Pharmacopoeia, heavy metal and pesticide residue standards. In a weight ratio of 3:6:10, CPF was boiled in six volumes of pure water for 60 minutes twice. The combined supernatants after filtration were vacuum‐dried at 60°C and reconstituted in DMSO (500 mg/mL) as stock and kept in 4°C.

### 2.3 Retroviral transduction and plasmid transfection

The mVenus‐p27K^−^ 20 was provided by Dr Toshihiko Oki (The University of Tokyo, Japan). It was mixed with packaging plasmids as described^18^ and transfected into 50% confluent HEK293T cells with a calcium phosphate precipitation method. The resulting lentiviral particles were used to infect A549 cells with 8 µg/mL polybrene. The infected cells with mVenus‐p27K^‐^ construct were selected by 0.2 mg/mL puromycin. The SPT16 (OHu22815D) and SSRP1 (OHu17195D) plasmid were obtained from GenScript Company and transfected into cells with Lipofectamine™ RNAiMAX (Invitrogen, Life Technologies).

### 2.4 SYBR green assay

Quiescent A549 (10 000 cells/well) and H1975 (7000 cells/well) cells were seeded in 96‐well plates. The same number of cells/well was kept as a baseline and stored at −80°C. After treatment, the medium was gently aspirated and replaced with 100 µL of lysis buffer as described.^18^ The cells were then lysed in the dark for 2 hours with shaking twice (5 min/each time). The frozen cells used as baselines were thawed at room temperature, lysed in the same buffer and transferred to the treatment plate. The fluorescence intensity of stained DNA was measured by a plate reader (FLUOstar Omega, BMG Labtech) with excitation at 485/20 nm and emission at 528/20 nm. GI value = [(FI of control − FI of baseline) − (FI of treated − FI of baseline)]/(FI of control − FI of baseline)×100%. FI = fluorescence intensity.

### 2.5 CCK‐8 assay

16HBE (10 000 cells/well) and BEAS‐2B (10 000 cells/well) were seeded in 96‐well plates overnight before the CPF treatment. The CCK‐8 reagent (Dojindo) was added into wells directly and measured by a plate reader (FLUOstar Omega, BMG Labtech) with the optical density (OD) at 450 nm. Survival rate (%) = OD_treated_/OD_control_ × 100.

### 2.6 Flow cytometry assay

To distinguish G_0_ and G_1_ cells, the cells incubated with Hoechst 33258 and Pyronin Y^21^ were injected into Gallios flow cytometer (Beckman Coulter). To determine mVenus‐p27K^‐^ fluorescence, the experimental cells were fixed with paraformaldehyde for 10 minutes at room temperature and washed with PBS prior to data acquisition using a flow cytometer (FACSCalibur II) equipped with CellQuest Pro software (BD Biosciences). Flow cytometry data were analysed using FlowJo software (version 10.0.8).

### 2.7 Quantitative reverse transcription‐PCR

Real‐time PCR was performed as described previously.^21^ The cDNA generated from total RNA (iScript™ cDNA Synthesis Kit, Bio‐Rad) was mixed with SYBR Green containing SensiMix™ Master Mix (Bioline). The reaction was subjected to a thermocycle at 95°C for 10 minutes followed by 45 cycles at 95°C for 15 seconds, at 60°C for 15 seconds and at 72°C for 15 seconds. The primer sequences are described in the supplementary information (Table S2).

### 2.8 Immunoblotting

A549 and H1975 cells were treated in 6‐well plates, and cell lysates were prepared as described ref.^19^. Primary antibodies against SPT16 (Cat. #: 12191), SSRP1 (Cat. #: 13421) and p27 (Cat. #: 3686) were from Cell Signaling Technology; Antibodies against SKP2 (Cat. #: sc7164) and c‐MYC (Cat. #: sc70469) were from Santa Cruz Biotechnology. GAPDH antibody (Cat. #: ab8245) was obtained from Abcam.

### 2.9 Chromatin immunoprecipitation (ChIP)

ChIP analysis was conducted with immunoprecipitation assay kit (Cat #: 17‐295, Merck) together with ChIP‐grade rabbit anti‐H3K4me3 (Cat #: ab8580, Abcam) and anti‐RNA polymerase II antibodies (Cat #: 664911, BioLegend) and isotype rabbit IgG (Cat #: sc‐2027, Santa Cruz Biotechnology). Immunoprecipitated DNA was quantified by quantitative real‐time PCR. See primer sequences in Table S3. ChIP assays were repeated thrice and calculated as fold enrichment relative to the control IgG and normalized by input DNA.

### 2.10 Immunocytochemistry

Cells detached from culture flasks were fixed in 10% buffered formalin solution, dehydrated and embedded in paraffin blocks. Selected samples were sectioned (5 mm thick) and stained with haematoxylin and eosin, Ki‐67 (Abcam, ab92353), as described previously.^22^ The primary antibodies were used at 1:500 for Ki‐67. The sections were finally mounted with DPX Mountant for histology analysis.

### 2.11 Imaging study

Nikon Ti‐E spinning disc confocal live cell microscope with a 20 × objective lens was used for time‐lapse imaging analysis of cells cultured in a 0.17‐mm glass‐bottom 6‐well plate (MatTek). An image was acquired every 15 minutes for 36 hours. Image acquisition and video conversion were performed with NIS‐Elements (version 4.5).

### 2.12 Chemical profiling of CPF extracts using LC‐MS/MS

Chemical profiling of CPF was performed on a Agilent 1290 LC System (Agilent Technologies) coupled with SCIEX TripleTOF 4600^®^ quadrupole time‐of‐flight mass spectrometry (AB Sciex) equipped with a DuoSpray Source. Chromatographic separation was achieved on an Acquity UPLC^®^ HSS T3 Column (2.1 × 100 mm i.d., 1.8 μm; Waters). The mobile phase consisted of water containing 0.1% formic acid (A) and acetonitrile (B). The following gradient condition was used: 0‐3.0 minutes, 5% B; 3.0‐7.0 minutes, 5% B–13% B; 7.0‐18.0 minutes, 13% B; 18.0‐19.0 minutes, 13% B–15% B; 19.0‐26.0 minutes, 15% B; 26.0‐30.0 minutes, 15% B–25% B; 30.0‐38.0 minutes, 25% B–65% B; 38.0‐43.0 minutes, 65%‐95% B; and 43.0‐45 minutes, 95% B. Column oven temperature was set at 30°C, while the flow rate was 0.3 mL/min. Ionization was conducted using an electrospray ionization (ESI) source. Data were collected under both positive and negative ion modes. The mass spectrometry was operated in full‐scan TOF‐MS (m/z 100‐1500) and information‐dependent acquisition (IDA) MS/MS modes; the collision energy was 40 ± 20 eV. Both ion source gas 1 and 2 were set 50 psi. Curtain gas was 35 psi. The temperature and ionspray voltage floating were 500°C and 5000/‐4500 V, respectively. Data recording and processing were performed by Analyst Ver. 1.6 software (AB Sciex).

### 2.13 Animals study

Male BALB/c nude mice (6 weeks old) and male ICR mice (4 weeks old) were maintained under specific pathogen‐free conditions with constant temperature (23 ± 2°C) and controlled light (12‐hour light: 12‐hour dark). To examine the effect of CPF on tumour growth, quiescent A549 cells were induced to re‐enter the cell cycle by plating at a low density and treated either with CPF at GI90 or with DMSO for 6 hours. The cell viability in both groups was evaluated by trypan blue exclusion, and they were >95% in both groups. Together with proliferative A549 cells, the pre‐treated cells, 1 × 10^7^ cells in 0.2 mL PBS, were subcutaneously injected into the left flank of BALB/c nude mice. The mice were anaesthetized for determining the tumour volume and weight 16 days after cancer cell injection.

### 2.14 Statistical analysis

The statistical software SPSS (version 18.0) was used for analysis. One‐way ANOVA was used to determine the difference between individual groups of data. Multiple comparison test was used to determine whether the difference between individual groups (*P* < .05) was significant.

## 3 RESULTS

### 3.1 CPF suppresses proliferative switch from G_0_ state in lung cancer cells

**Figure 1:**
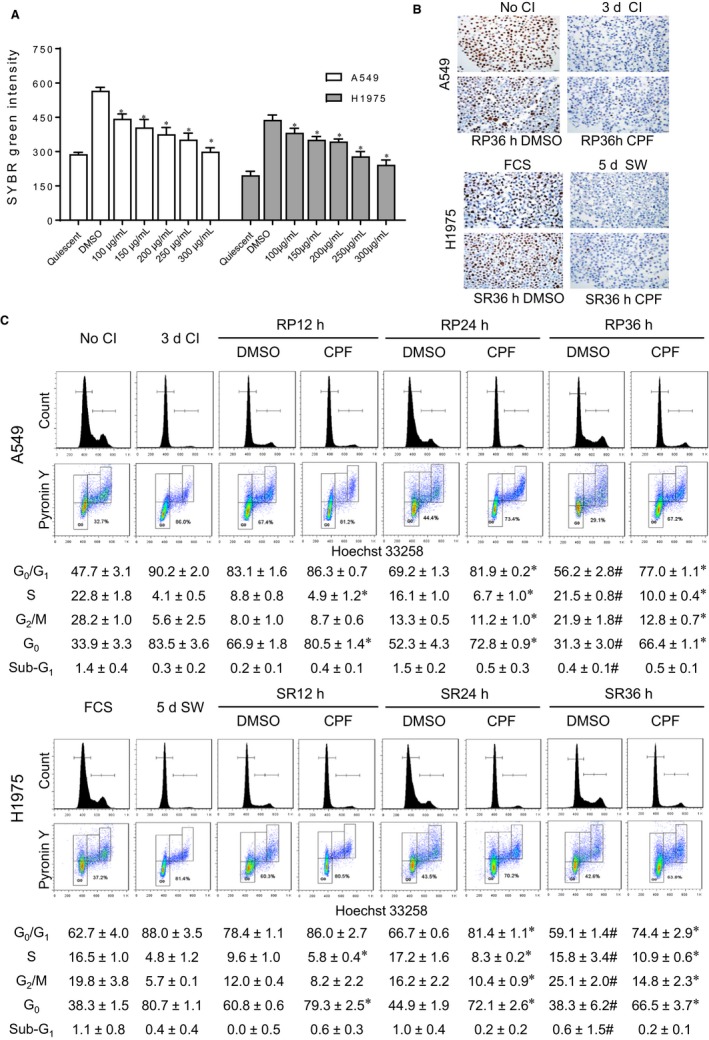
CPF suppresses proliferative switch from G_0_ state in lung cancer cells. A, CPF or DMSO was administered upon releasing from quiescence for 36 h. The treated two lung cancer cell lines together with the quiescent baseline cells were incubated with lysis buffer containing SYBR Green, and the DNA contents were measured. *P < .05 vs DMSO. B, The non‐quiescent and quiescent cells and the cells treated with DMSO or CPF at GI90 upon cell cycle re‐entry for 36 h were harvested for analysis of Ki‐67 by immunocytochemical staining. No CI: no contact inhibition; 3d CI: contact inhibition for 3 d; RP36h DMSO: treatment of replated cells with DMSO; RP36h CPF: treatment of replated cells with CPF at GI90. FCS: no removal of foetal calf serum; 5d SW: serum withdrawal for 5 d; SR36h DMSO: treatment of serum‐replenished cells with DMSO; SR36h CPF: treatment of serum‐replenished cells with CPF at GI90. C, The non‐quiescent cells, quiescent cells and cells after releasing from G_0_ at indicated time were stained with Hoechst 33258 and Pyronin Y. Representative images and quantification data after analysis of Hoechst 33258 alone or both Hoechst 33258 and Pyronin Y are shown. Data are expressed as the mean ± SD from three experiments. *P < .05 vs DMSO at each time‐point. #*P* > .05 vs No CI or FCS

As described previously,^18^ experimental quiescence was achieved by removing serum from culture media for 5 days in the lung cancer cell line H1975 or contact inhibition for 3 days in the lung cancer cell line A549. Cell cycle re‐entry was rendered by replenishing serum in serum‐deprived cells or replating the contact‐inhibited cells at lower density. By comparing DNA content immediately before cell cycle re‐entry (ie at quiescence), the release from quiescence led to a surge of DNA synthesis at 36 hours (Figure 1A). CPF treatment, which was introduced at the time when the cells were released from quiescence, suppressed DNA synthesis in a dose‐dependent fashion. The concentrations of CPF at which the cytostatic action (ie antiproliferation) reached 50% (GI50) and 90% (GI90) were calculated (Table S4 **)**. The inhibitory effect of CPF at GI90 for 36 hours on proliferative switch was verified by Ki‐67 immunostaining (Figure 1B).

To determine the effect of CPF on cell cycle phase distribution, quiescent A549 and H1975 cells were induced to re‐enter the cell cycle in the presence of CPF at GI90. The cells were harvested at 12‐hour intervals and subjected to flow cytometric analysis. In comparison with control cells, there was a clear increase in the proportion of cells at G_0_/G_1_ and decrease in S and G_2_/M phase following CPF treatment at 24 hours following cell cycle re‐entry (Figure 1C). Since cells at G_0_ and G_1_ are both diploid, we used a double staining method to quantify the G_0_ fraction.^21^ The reduced G_0_ fraction seen in controls as early as 12 hours following release from quiescence was prevented to a large extent by CPF (Figure 1C). In control cells, a complete return of cell cycle distribution to proliferative state was achieved at 36 hours following release from quiescence (all *P* > .05 compared with No CI and FCS). However, treatment with CPF caused delay in the re‐entry in both cell lines, as the distribution of all cell cycle phases remained different significantly from control at 36 hours (Figure 1C). No statistically significant change in cell viability was observed on treatment with CPF at GI90 as cell cycle analysis showed <2% cell population in the sub‐G_1_ fraction.

**Figure 2:**
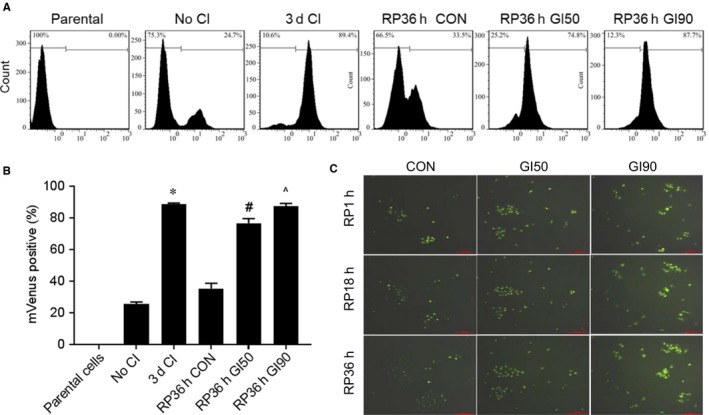
CPF retains the mVenus‐p27K^‐^ signal upon cell cycle re‐entry. A, Flow cytometric analysis of mVenus fluorescence in A549 parental cells, mVenus‐p27K^−^ without contact inhibition, and contact‐inhibited mVenus‐p27K^−^, without or with CPF upon release from quiescence. B, Quantification data are expressed as the mean ± SD from three experiments. *P < .05 vs No CI; #*P* < .05 vs RP36h CON; and ^*P* < .05 vs RP36h GI50. C, Images of A549‐mVenus‐p27K^‐^ cells released from quiescence with or without CPF

To further validate the CPF action, we transduced A549 cells with mVenus‐p27K^−^ plasmid. This mutant p27 cannot bind cyclin‐dependent kinase but maintains an intact domain for ubiquitination. CPF was introduced when mVenus‐p27K^−^ cells were released from quiescence following 3‐day contact inhibition. There was a significant increase in fluorescent signal over the course of contact inhibition compared to the proliferating cells (Figure 2A,B). The signal was reduced upon cell cycle re‐entry. However, treatment with CPF at GI50 and GI90 led to a clear retention of mVenus‐p27K^−^ signal compared with vehicle‐treated control (Figure 2C and Video). Taken together, these data underscore that CPF can impede cell cycle re‐entry of quiescent lung cancer cells.

### 3.2 CPF treatment decreases FACT mRNA and protein levels and transcriptional activity

**Figure 3:**
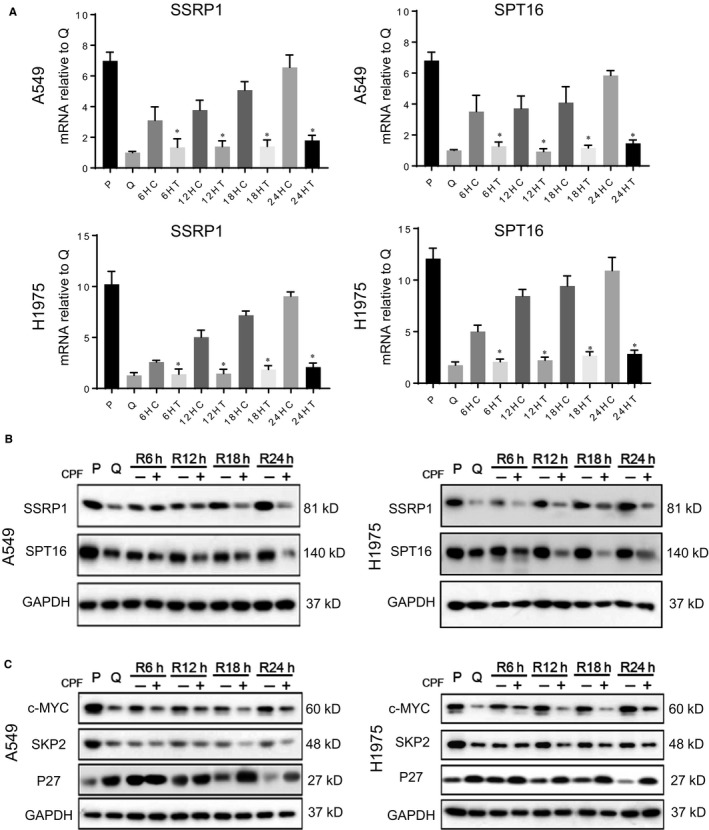
CPF treatment decreases FACT mRNA and protein levels. Upon release from quiescence, A549 and H1975 cells were treated with CPF at GI90 for 6‐24 h and then harvested for RT‐qPCR (A) or immunoblotting (B) for SPT16 and SSRP1. P: proliferative cells, Q: quiescent cells; R: release from quiescence; C: control; and T: CPF treatment. *P < .05 vs control at same time‐point. C, The quiescent cells were exposed to CPF at GI90 for the indicated time from releasing from quiescence and analysed for p27 and its degradation proteins by immunoblotting. *P < .05 vs control at same time‐point

We have shown recently that FACT is required in cell cycle re‐entry by quiescent lung cancer cells.^18^ To establish the mechanism by which CPF impedes cell cycle re‐entry, we examined the impact of CPF on mRNA and protein levels of FACT in cell cycle re‐entry. Treatment with CPF efficiently reduced the surged mRNA (Figure 3A) and protein (Figure 3B, Figure S2A) levels of FACT subunit SSRP1 and SPT16 over the time period of 6‐24 hours compared with vehicle control cells. Moreover, consistent with our previous finding that FACT promotes cell cycle re‐entry via p27 and its regulatory proteins including c‐MYC and SKP2,^18^ the CPF treatment reduced c‐MYC and changed SKP2 and p27 over the same period (Figure 3C, Figure S2B).

**Figure 4:**
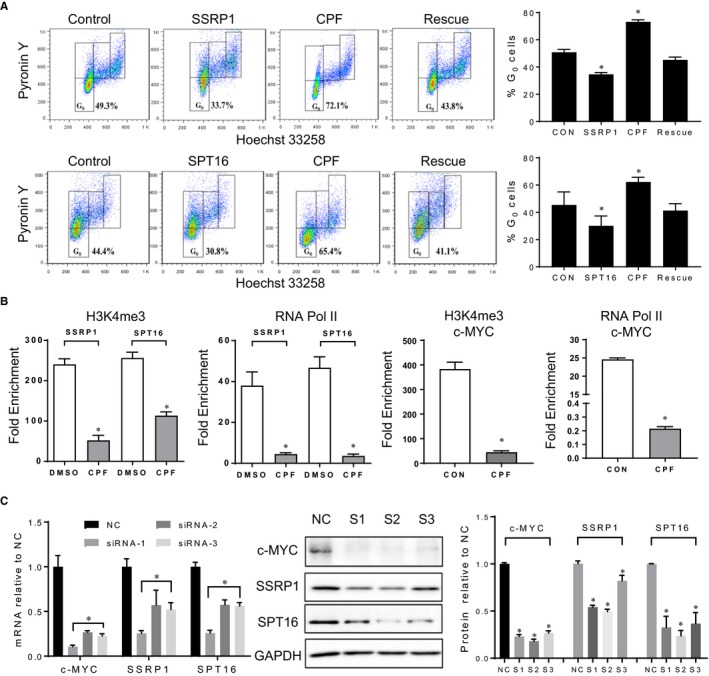
CPF suppresses transcription of FACT and c‐MYC genes. A, Quiescent A549 cells were transfected with either empty vector (control), or FACT plasmid (SPT16+ or SSRP1+), or treated with CPF at GI90, or a combination of FACT plasmid and CPF at GI90 (rescue) upon cell cycle re‐entry for 36h. The cells were harvested for cell cycle analysis. *P < .05 vs control; B, ChIP was performed in the presence of CPF at GI90 or DMSO for 36 h following release from 3‐day contact inhibition in A549 cells. PCR primers were designed based on FACT and c‐MYC promoter or control region. *P < .05 vs control. C, Knockdown of c‐MYC with three sets of siRNA and its impact on FACT mRNA and protein levels by RT‐PCR, immunoblotting and quantification. *P < .05 vs NC control

To verify that the CPF‐led inhibition of cell cycle re‐entry is mediated by reduction of FACT mRNA and protein levels, we transfected quiescent A549 cell line with an expression vector containing SSRP1 or SPT16 for 4 hours after release from quiescence. At 36 hours, the ectopic expression of either FACT subunit significantly reduced the G_0_ fraction compared to control. While CPF alone increased the cells at G_0_ fraction compared to control, the effect of CPF on G_0_ fraction was diminished when FACT subunits were overexpressed simultaneously (Figure 4A, Figure S2C). Hence, CPF action on cell cycle re‐entry could be mediated by its impact on FACT. We then used ChIP assays to determine the effect of CPF on H3K4 tri‐methylation and RNA polymerase II occupancy at FACT gene promoters in A549 cell line. There was a significant reduction of H3K4 tri‐methylation and RNA polymerase II recruitment at SSRP1 and SPT16 gene promoter regions in the presence of CPF compared with vehicle control (Figure 4B).

Considering that the CPF treatment reduced c‐MYC almost at same time as FACT, we used ChIP assays to determine the occupancy of H3K4 tri‐methylation and RNA polymerase II at c‐MYC gene promoter in the presence of CPF. Indeed, compared with vehicle control, CPF treatment led to a significant reduction of transcriptional activity at c‐MYC promoter (Figure 4B) and mRNA levels (Figure S2D). While it is clear that FACT can regulate c‐MYC in lung cancer cells,^18^ whether or not c‐MYC can influence FACT expression in lung cancer cells remains unknown. Hence, we used siRNA to knockdown c‐MYC and observed a clear decrease in SSRP1 and SPT16 mRNA and protein levels (Figure 4C). These data indicate the presence of a positive feedback loop between FACT and c‐MYC expression in lung cancer cells and suggest that CPF can suppress transcription of FACT and c‐MYC either directly or indirectly through their interconnected positive loop.

### 3.3 Quiescent lung cancer cells exposed to CPF in vitro reduced growth in vivo

**Figure 5:**
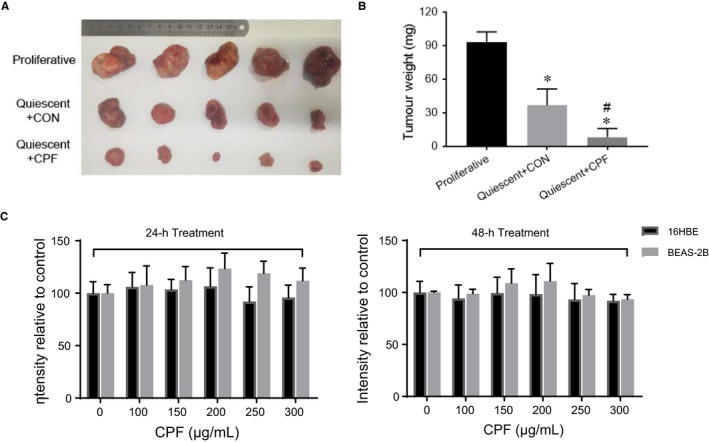
Quiescent lung cancer cells exposed to CPF in vitro reduced growth in vivo. Quiescent A549 cells were induced to re‐enter the cell cycle by plating at a low density and treated either with CPF at GI90 or with DMSO for 6 h. Together with proliferative A549 cells, the pre‐treated cells were subcutaneously injected into the left flank of 6‐week‐old male BALB/c nude mice (1 × 10^7^ cells per mouse). Tumour volume (A) and weight (B) on day 16 of experimentation were shown. *P < .05 compared with the proliferative group; #*P* < .05 compared with the DMSO pre‐treated control. C, The response of normal human bronchial epithelial cells to CPF for indicated dose and duration was analysed by CCK‐8 reagent

Study in vivo of cell cycle re‐entry by quiescent cancer cells is difficult as it is not possible to synchronize cancer cells at G_0_ in living organisms. Hence, A549 lung cancer cells were induced to quiescence by contact inhibition for 3 days. These cells were then released in the presence or absence of CPF at GI90 for 6 hours. While the cells remained at G_0_ based on cell cycle phase analysis, A549 cells were subcutaneously injected into nude mice. To demonstrate the difference between quiescent and proliferative state, the same number of non‐synchronized A549 cells was included. The proliferative and quiescent cells formed measurable tumours at 3.00 ± 0.89 days and 6.80 ± 1.33 days, respectively. The cells treated with CPF in vitro exhibited delayed tumour formation at 12.20 ± 1.72 days. By day 16, the growth of proliferative cells reached an ethical end‐point. In contrast, CPF‐treated cells developed smaller tumours (Figure 5A,B) and were significantly lighter compared with tumours from vehicle‐treated cells. These data indicate that a 6‐hour in vitro exposure of quiescent lung cancer cells to CPF at GI90 can reduce tumour formation over 16 days mostly likely via inhibiting cell cycle re‐entry as illustrated under in vitro condition. The same dose of CPF had no significant impact on normal bronchial epithelial cells (16HBE and BEAS‐2B) in vitro (Figure 5C).

## 4 DISCUSSION

The histone chaperone FACT contributes to the maintenance of a flexible chromatin landscape. By removing and presenting histones to naked DNA, FACT affects selectively nucleosome disassembly and assembly thereby regulating transcription and elongation of a small fraction of genes.^15^ Hence, a therapy targeting FACT can influence neither globally nor on FACT only. This multitarget approach, in theory, should be beneficial considering the ability of cancer cells to acquire resistance to single‐target therapies. FACT is highly expressed in poorly differentiated tumours with unfavourable outcome.^15^ Reducing expression of either FACT subunit leads to inhibition of proliferation and tumour cell death, probably due to the mechanism that FACT selectively promotes transcription of genes that stimulate proliferation and prohibit cell death and differentiation.^16^, ^17^ We have shown recently that FACT is necessary and sufficient in the switch from G_0_ to the proliferative state in lung cancer cells.^18^ FACT protein levels are low at G_0_ compared to the proliferating state but quickly surge upon cell cycle re‐entry. Knockdown of FACT hindered cell cycle re‐entry of quiescent cells, likely through a reduction in c‐MYC gene expression for several reasons. Firstly, FACT binding at c‐MYC promoter is enriched significantly. Secondly, knockdown of either FACT subunit decreases c‐MYC mRNA and protein levels. Thirdly, knockdown of FACT also reduced c‐MYC target gene SKP2 mRNA and protein levels, resulting in an increase in G_0_ maintaining p27 protein levels. The present study has taken findings one step further by providing evidence that the FACT and c‐MYC may form a positive loop in promoting each other's gene expression. We found that knockdown of c‐MYC can decrease FACT subunit SPT16 and SSRP1 mRNA and protein levels. Hence, consistent with previous reports in neuroblastoma^23^ and fibrosarcoma^24^ cell lines that FACT can be a c‐MYC target gene, a reverse regulation of FACT by c‐MYC is also present in lung cancer cells. Indeed, the MYC E‐box transactivation motif can be found within ~500 base pairs upstream of transcriptional start sites of SPT16 and SSRP1.^23^ Establishing this positive loop is important as c‐MYC is known for its critical role in regulation of the G_0_ maintenance proteins p27 and SKP2,^21^, ^25^, ^26^ whereas FACT is a newly emerging player in the regulation of cell cycle re‐entry.^18^ Functioning as a histone chaperone protein and key transcriptional factor, respectively, the mutual influence of FACT and c‐MYC is expected to be broad and well beyond each other. The positive loop between FACT and c‐MYC can be considered as a therapeutic target in preventing recurrence of lung cancer, since, despite the significant progress in primary treatment of lung cancer, recurrence still occurs after the molecular targeted treatment and immunotherapy.^27^, ^28^

While anticancer drugs such as doxorubicin, cyclophosphamide and cisplatin are able to target cancer cells in the non‐proliferating state, their genotoxicity and clinical toxicity make them unsuitable for long‐term therapy in cancer survivors. Thus, any intervention as part of post‐treatment care ought to have different characteristics from the intensive, short‐term, primary treatment. Based on the three selection criteria of effective therapy, minimal side‐effect profiles and financially affordable treatment, and the notion of ‘old drugs for new uses’, we screened classical Chinese herbal medicines from the book ‘Treatise on Miscellaneous Diseases’.^19^ CPF outcompetes others and meets the selection criteria. Through physical (contact inhibition) and chemical (serum removal) induced quiescence and quantification of G_0_ fraction and mVenus‐p27 intensity, CPF showed a clear capacity to impede proliferative switch from quiescent state. The action of CPF is rather quick as a transcriptional reduction of FACT and c‐MYC was noted 6 hours following administration upon release from quiescence. Furthermore, the impact of CPF on quiescent lung cancer cells appears to be relatively long‐lasting, as a 6‐hour exposure to CPF in vitro can result in reduction of tumour size and weight 16 days following transplantation in vivo. Due to the lack of animal models to simulate the process of cell cycle re‐entry by G_0_ cancer cells, the effect of CPF administered in living organism remains to be determined. To link the structure with impeding cell cycle re‐entry action, we embarked on a process of isolating active compound from CPF. Of the three components of CPF, the impeding cell cycle re‐entry action is derived from *Coptis chinensis,* and not *Pinellia ternata* or *Fructus trichosanthis*. We will screen the identified 43 compounds from *Coptis chinensis,* and, if they are not the major active ingredients, we will use HPLC to obtain the fraction of *Coptis chinensis* and test each fraction in our platform of cell cycle re‐entry. The effective fraction will be used for isolation of the active compound, which will then be validated by comparing its action and mode of action with CPF and *Coptis chinensis*.

The presented work also reflects our effort to use modern research tools to develop a system to scientifically determine the efficacy of ancient Chinese medicine recipes. In 2015, the Chinese scientist Youyou Tu was awarded the Nobel Prize for the development of an antimalarial drug extracted from Artemisia annua L.^29^ Realgar‐Indigo naturalis receipt and its ingredients have been proven to be effective in treating human acute promyelocytic leukaemia.^30^ Although these are evidences of the presence of effective compounds in traditional Chinese medicines, for most Chinese medicine receipts the exact action and mode of action are not well defined. Since a great population is using traditional medicine,^31^ it is necessary to evaluate and validate the biomedical potential of Chinese medicine so that evidence can be provided for each recipe for its disease indication, molecular target and active ingredients.

## CONFLICT OF INTEREST

The authors declare no competing interests.

## AUTHOR CONTRIBUTIONS

LB, CX, LJ, SJ, SH, MY, YW, QW, GG, YW, XS and YK conducted experiments, analysed data and wrote the manuscript. XZ, PD, TL and JZ supervised research, interpreted data and wrote the manuscript. LX and QD designed the study.

## ETHICS APPROVAL AND CONSENT TO PARTICIPATE

The animal study was approved in Sino‐British SIPPR/BK Lab Animal Ltd (animal authorization reference number: SCXK2013‐0016) and performed in accordance with the Declaration of Helsinki.

## Data Availability

The original data of this study are available from corresponding author upon request.
