# Supplementary figures and images for: CPF impedes cell cycle re‐entry of quiescent lung cancer cells through transcriptional suppression of FACT and c‐MYC
Source: J Cell Mol Med. 2020 Jan 20;24(3):2229–39. doi: 10.1111/jcmm.14897 (PMC7011132; doi:10.1111/jcmm.14897)

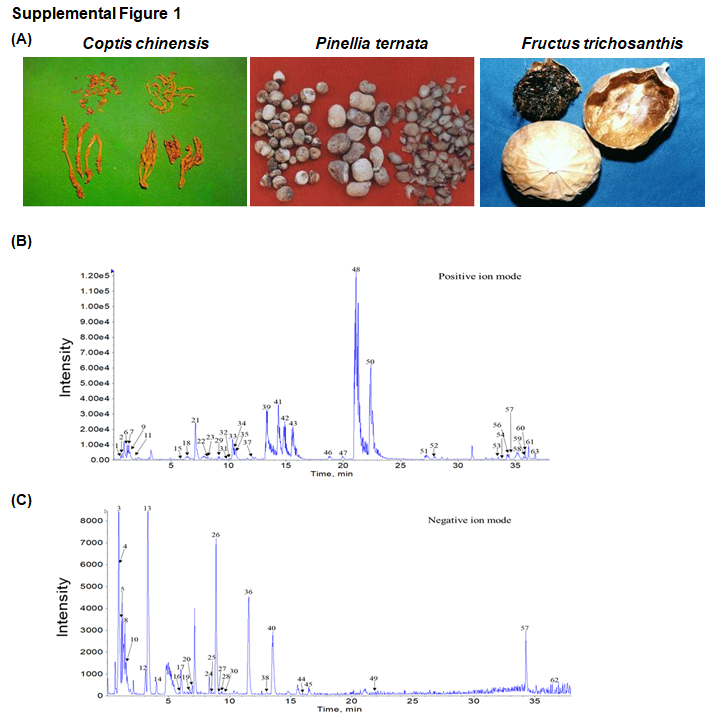

Supplement: Supplementary file 1 [file JCMM-24-2229-s001.tif]

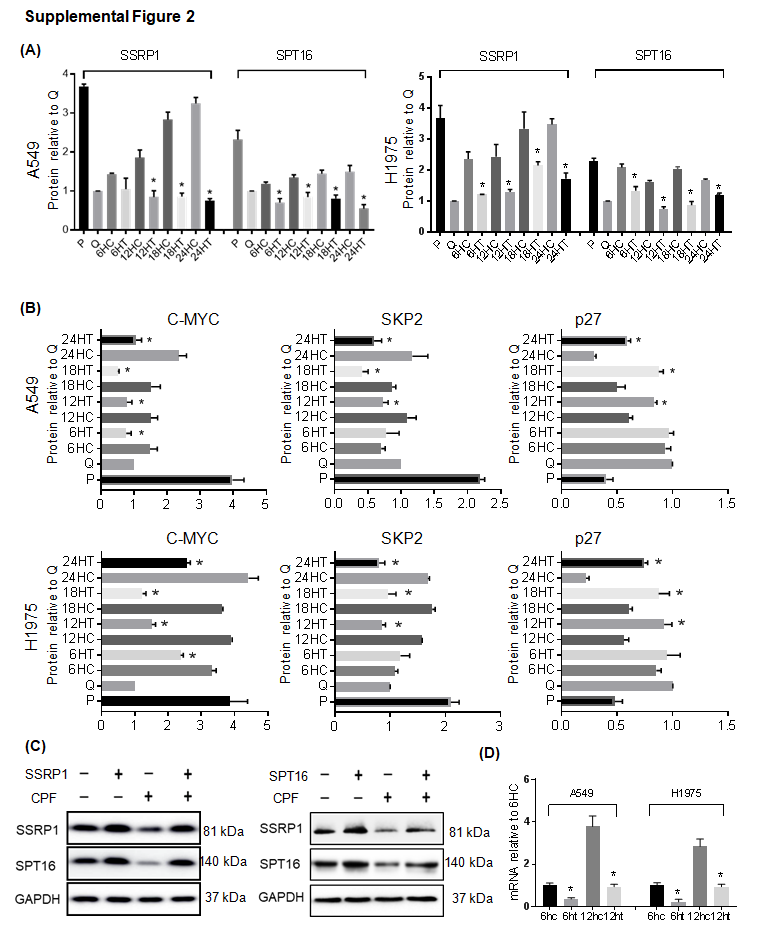

Supplement: Supplementary file 2 [file JCMM-24-2229-s002.tif]
